# The Effect of Genetic and Environmental Variation on Genital Size in Male *Drosophila*: Canalized but Developmentally Unstable

**DOI:** 10.1371/journal.pone.0028278

**Published:** 2011-12-08

**Authors:** Austin P. Dreyer, Alexander W. Shingleton

**Affiliations:** Department of Zoology/Ecology Evolutionary Biology and Behavior Program, Michigan State University, East Lansing, Michigan, United States of America; University of Otago, New Zealand

## Abstract

The genitalia of most male arthropods scale hypoallometrically with body size, that is they are more or less the same size across large and small individuals in a population. Such scaling is expected to arise when genital traits show less variation than somatic traits in response to factors that generate size variation among individuals in a population. Nevertheless, there have been few studies directly examining the relative sensitivity of genital and somatic traits to factors that affect their size. Such studies are key to understanding genital evolution and the evolution of morphological scaling relationships more generally. Previous studies indicate that the size of genital traits in male *Drosophila melanogaster* show a relatively low response to variation in environmental factors that affect trait size. Here we show that the size of genital traits in male fruit flies also exhibit a relatively low response to variation in genetic factors that affect trait size. Importantly, however, this low response is only to genetic factors that affect body and organ size systemically, not those that affect organ size autonomously. Further, we show that the genital traits do not show low levels of developmental instability, which is the response to stochastic developmental errors that also influence organ size autonomously. We discuss these results in the context of current hypotheses on the proximate and ultimate mechanisms that generate genital hypoallometry.

## Introduction

Within a population or species, variation in body size is expected to be accompanied by approximately equivalent variation in the size of individual morphological traits. Such covariation is necessary to maintain correct body proportion across the range of body sizes observed in animal populations. A notable exception to this pattern, however, is the relationship between genital size and body size in arthropods. The genitalia of most arthropods are more-or-less the same size in both large and small individuals [1], [2], [3], [4], [5]. Consequently, smaller males have proportionally larger genitalia than larger males. While the phenomenon is most obvious in males, it has also been observed in female arthropods [5], [6], [7], [8], as well as some mammals [9], [10], [11].

The scaling relationship between two traits among individuals of the same developmental stage in a population is called a static allometry, and is typically described using the allometric equation, 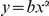, where *x* and *y* are the size of two traits [12]. Log transformation of this equation produces the simple linear equation 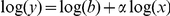, and log-log plots of the size of different traits among individuals in a population typically reveal linear scaling with a slope of *α*, called the allometric coefficient [12]. When 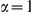, the relationship between *x* and *y* is called isometry, with the ratio of *y* to *x* remaining constant across a range of *x*. When 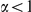 or 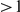 the relationship is hypo- or hyperallometric, respectively, with relative size of *y* decreasing (hypoallometry) or increasing (hyperallometry) with an increase in *x*. Fundamental to the concept of allometry is that x and y covary; that is the factors that generate variation in *x* also generate variation in *y*. The allometric coefficient therefore captures the extent to which these factors affect *y* relative to *x*. If a factor that generates size variation affects both traits equally, then *y* will scale isometrically to *x* (assuming that all size variation is due to the factor). If the factor has a lesser or greater effect on *y* than *x*, *y* will scale with *x* hypo- or hyperallometrically respectively [13].

The observed hypoallometry of the male genitalia in arthropods suggests that genital traits are relatively insensitive to the factors that generate size variation among individuals in a population. Size variation may be generated by environmental variation (plasticity), genetic variation and developmental instability (variation due to stochastic developmental perturbations within an individual, [14], [15], [16]). Consequently, we might expect the genitalia to be environmentally canalized, genetically canalized and/or developmentally stable. Here, we define canalization as the property of a trait to resist genetic or environmental variation [17], [18], [19], and developmental stability as the property of a trait to resist stochastic developmental perturbations that generate fluctuating asymmetry (FA) in a bilaterally symmetrical organism [19], [20]. Previous studies have demonstrated that the male genitalia of *Drosophila* are environmentally canalized, at least with respect to developmental nutrition, temperature and larval crowding [21]. It is unclear, however, whether they are also genetically canalized and developmentally stable.

In contrast to our lack of understanding of the developmental mechanisms that underlie genital hypoallometry, there are a number of hypotheses as to its adaptive significance [22], [23], [24]. A general theme of many of these hypotheses is that there is stabilizing selection on male genital size, either because females are physically unable to mate with males bearing inappropriately-sized genitalia [25] or because females prefer males with genitalia of a specific size [5]. Alternatively, hypoallometry may arise because there is directional selection on increased genital size that is strong in small males but weak or absent in large males [26]. These different hypotheses, while not mutually exclusive, serve to emphasize the observation that the form of selection on genitalia can be difficult to infer from patterns of allometry [27].

Elucidating the proximate mechanisms that generate genital hypoallometry may help clarify the ultimate evolutionary processes that cause it. This is because different evolutionary hypotheses suggest different patterns of genetic and environmental variation in genital size. For example, if genital hypoallometry were a consequence of elevated levels of stabilizing selection on genital size, we would expect to see a reduction in the level of genetic variation in genital size relative to other traits: that is they should be genetically canalized [10], [28]. We might also expect the genitalia to be environmentally canalized and developmentally stable [28], [29], [30], [31], [32], [33], but see [34].

Here we measure the level of genetic variation and developmental stability in genital and somatic traits in *D. melanogaster*. Consistent with our understanding of the mechanisms that generate morphological scaling relationships, we find that genital traits are genetically canalized. However, the genitalia are only canalized with respect to genetic factors that affect the size of all organs in the body systemically. Genital traits are not canalized with respect to genetic factors that affect the size of individual organs autonomously. Further, we find that genital traits are not developmentally stable as indicated by elevated levels of fluctuating asymmetry relative to some somatic traits. We discuss these findings in light of current theories of genital evolution and argue that stabilizing selection on genital size alone is insufficient to explain their hypoallometric relationship with body size.

## Materials and Methods

### Fly Stocks

Male flies were from 38 of the Core40 isogenic wild-type lines from the *Drosophila* Genetic Reference Panel (DGRP).

### Fly Rearing

#### 1) Genetic Variation

Genetic variation was assayed among the Core40 isogenic DGRP lines. Larvae from each line were reared in vials at low density (≤50 larvae) on standard cornmeal∶molasses medium at 25°C in constant light. We collected, dissected and measured males from at least three vials per line, totaling ten males per line.

#### 2) Developmental Instability

We used three of the Core40 DGRP lines to assay developmental instability (lines 303, 324, 335). Larvae were reared at low density (≤50 larvae) in ten vials per line, as described above. We selected, dissected and measured five males from each vial, totaling 50 males per line.

### Morphology

Five organs were dissected from each male fly: three somatic traits (the wing, the femur of the first leg, and the maxillary palp) and two genital traits (the posterior lobe of the genital arch and the anal plate). Organs were mounted in dimethyl hydantoin formaldehyde for imaging. Organ measurements were taken as area for the wing, the maxillary palp, the posterior lobe of the genital arch, and the anal plate and as the length of the femur, using a Leica DM6000B compound microscope and Retiga 200R digital camera. We also measured a fourth somatic trait, thorax length, as the distance between the attachment of the neck to the posterior tip of the scutellum using a Leica MZ16FA dissecting microscope and a Leica DFC250 digital camera. Images were analyzed using ImagePro. All linear measurements were squared prior to analysis to convert them to the same dimension as area measurements. All data were then natural log transformed to allow the fitting of the linear allometric equation. For the measurement of FA, we measured the wing, femur, maxillary palp and posterior lobe of the genital arch from both sides of the fly three times, and calculated measurement error using the methods of Palmer and Strobeck [35].

### Analysis

#### 1) Genetic Variation

We fit the data to the following linear model:
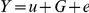
where *Y* is the morphological measurement, *u* is the intercept term, *G* is the effect of line (random factor) and *e* is remaining non-genetic variation. We used the *lmer* function in the *lme4* package in R [36] to extract the variance components using REML for *G*, which is a measure of the total genetic variation of *Y*, here referred to as *V_T_*. Each *V_T_* was then converted into a coefficient of variation (*CV_T_*) using the formula 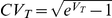 [37]. *CV_T_* was used as a measure of a trait's total genetic variation.

We reanalyzed the data but statistically controlled for variation in other traits by including them as covariates in our model. This allowed us to estimate the amount of genetic variation in a trait that was orthogonal to and independent of variation in all other traits, that is a trait's organ-autonomous genetic variation. The final model was:
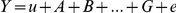
where *A*, *B etc.* are the size of all other traits. We then extracted the variance components for *G*, (*V_I_*) which is a measure of the organ-autonomous genetic variation of *Y*, here referred to as *V_I_*, from the analysis. We used *V_I_* to calculate the organ-autonomous coefficient of variation (*CV_I_*) using the formula 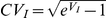.

Each dataset was sampled with replacement to generate 1000 bootstrap datasets, which were analyzed and used to construct a 95 percent confidence interval of each trait's total (*CV_T_*) and organ-autonomous (*CV_I_*) genetic variation.

#### 2) Genetic Static Allometry

The allometric coefficient of the genetic static allometry (where size variation is solely a consequence of genetic variation) was calculated from the mean log-transformed trait measurements for each line. We used these data to calculate the variance-covariance matrix for traits among lines, and extracted the first eigenvector from this matrix using the *svd* function in the base package of R [36]. The allometric coefficient is reflected by the loadings of the first eigenvector. Isometry occurs when all loadings of the vector equal 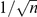, where *n* is the number of traits measured. Multiplying the loadings by 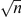 gives the bivariate allometric coefficient for each trait against a measure of overall body size. We used a random-variable bootstrap method to generate 95 percent confidence intervals for the allometric coefficients for each trait [21].

#### 3) Developmental Instability

Here we define developmental instability as the imprecision that results from developmental noise, the random developmental processes that cause a trait to deviate from its expected growth trajectory given its genotype and environment. Conversely, the capacity of the growing organ to counteract developmental noise is defined as developmental stability. Fluctuating asymmetry is therefore a measure of developmental instability, and is reduced in organs that are developmentally stable. We used the FA10b index, which corrects for measurement error, to quantify fluctuating asymmetry for the wing, maxillary palp, femur and posterior lobe of the genital arch [35]. To calculate FA10b we fit the repeated measurement of each trait to the following model:
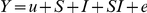
where *u* is the intercept term, *S* is the effect of body side, left or right (fixed factor), *I* is the effect of the individual (random factor), *SI* is the interaction between individual and side and *e* is measurement error. We used the *lmer* function in the *lme4* package in R [36] to estimate the variance components for *SI* (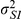), which is used to calculate FA10b:
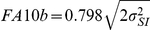



We used the *MCMCglmm* function in the *MCMCglmm* package in R [36] to generate values of 95 percent support for each trait's level of fluctuating asymmetry. We used a prior equal to the variation in wing size measurements to generate parameter estimates and compared the results to those using a non-informative prior and found no difference in parameter estimates.

All traits were tested for antisymmetry and directional asymmetry by assaying the distribution of trait size on the right (R) and left (L) side of an individual. For antisymmetry we tested the (R – L) distribution for normality and for directional asymmetry we compared the mean of the signed (R – L) to zero [35]. (R – L) for almost all traits was normally distributed (Shapiro-Wilk test, *P*>0.004 with Bonferonni correction). The only exceptions were the maxillary palps of line 303 and wings of line 335 (P<0.004 for both). Plotting R versus L for the size of both these traits suggested three maxillary palp measurements from line 303 and seven wing measurements from 335 were outliers. Removal of these data normalized the distribution of (R – L) for both these traits, although their inclusion had no effect on the analysis (not shown). The maxillary palp of line 324 also showed evidence of slight directional asymmetry with mean (R – L) deviating significantly from zero (t-test, p<0.004 with Bonferoni correction). However, the mean (R – L) was less than *FA*4*a* (where 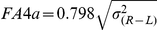, [35]), and so any directional asymmetry was considered to be a consequence of developmental instability [35].

## Results

**Figure 1:**
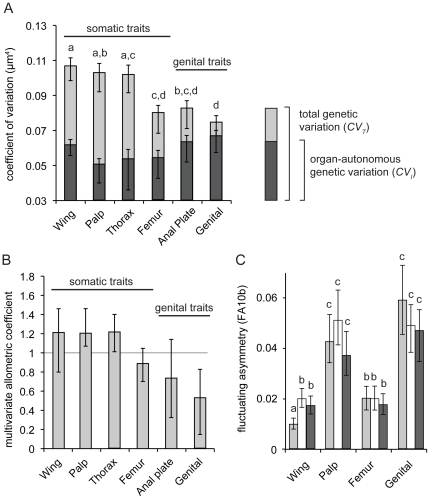
Genetic variation, allometric coefficient and fluctuating asymmetry of somatic and genital traits in male *Drosophila melanogaster*. (A) Genital traits had low levels of total genetic variation (light gray bars, *CV_T_*) but not low levels of organ-autonomous genetic variation (dark gray bars, *CV_I_*). The difference between total genetic variation and organ-autonomous variance is an estimate of genetic variation that is correlated with variation in other traits (‘systemic’ genetic variation). Columns with the same letter are not significantly different for total genetic variation (*CV_T_*) using Tukey's HSD (*P>*0.05). Traits do not differ for organ-autonomous genetic variation (*CV_I_*) using Tukey's HSD (*P*>0.05 for all) (B) The low systemic genetic variance of the genital traits reflected their low multivariate allometric coefficient compared to most somatic traits, although these differences are not significant for multiple comparisons (Tukey's HSD, *P*>0.05 for all). Grey horizontal line is isometry. (C) Genital traits did not show low levels of fluctuating asymmetry. Light grey bars, line 303, white bars, line 324, dark grey bars, line 335. Within a line, columns with the same letter are not significantly different for FA using Tukey's HSD (*P* 0.05). All error bars are 95% confidence intervals.

The total amount of genetic variation (*CV_T_*) was lower for the genital traits (genital arch and anal plate) than for the somatic traits (wing, maxillary palps and thorax) (Fig. 1A), although when correcting for multiple comparisons this reduction in genetic variation was significant only for the genital arch (Tukey's HSD, p<0.05). In contrast, none of the traits differed in their level of organ-autonomous genetic variation (*CV_I_*) – that is the amount of genetic variation in trait size that is not correlated with variation in the size of other traits – when correcting for multiple comparisons (Tukey's HSD, p>0.05) (Fig. 1B. These results suggest that very little of the variation in genital trait size is a response to variation in genetic factors that affect all traits systemically. It is the response to these systemic genetic factors that controls the slope of an organ's scaling relationship with body size on a genetic static allometry: traits with low response should scale hypoallometrically with body size. Correspondingly, we found that the genital traits were significantly more hypoallometric to overall body size than most somatic traits (Fig. 1B). Interestingly, the femur of the first leg, like the genital traits, displayed both low levels of genetic variation and scaled more hypoallometrically to overall body size than other somatic traits (Figs. 1A & B).

Although the genital traits were genetically canalized with respect to factors that affect organ size systemically, they did not show low levels of developmental instability. In contrast, within each of the three lines examined, the maxillary palp and the genital arch had significantly higher levels of fluctuating asymmetry than either the wing or the femur (Tukey's HSD p>0.05) (Fig. 1C).

## Discussion

Elucidating the causes of the unusual scaling relationship between genital size and body size in arthropods is an active but unresolved area of research [10], [27], [38], [39], [40]. The goal of our study was to begin to explore the proximate mechanisms that underlie genital hypoallometry, specifically the response of male genital size to genetic variation and to stochastic developmental errors.

The slope of a scaling relationship between body and organ size captures the extent to which factors that generate variation in body size also generate variation in organ size (and vice versa). Consequently, traits that scale hypoallometrically to body size, such as the genitalia, are expected to show low levels of variation in response to genetic and environmental factors that affect both body and organ size. Previous studies have shown that, as expected, genital traits show low levels of variation in response to environmental factors that affect body and organ size; the genitalia are thus environmentally canalized [21]. Our data show that genital traits also show low levels of variation in response to genetic factors that affect body and organ size, that is the genitalia are genetically canalized (Fig. 1A). Importantly, however, genital traits do not show low levels of variation in response to genetic factors that autonomously affect their size (Fig. 1A). These genetic factors presumably affect organ size at the level of individual organs and not through systemic mechanisms.

The genitalia also do not appear to show low levels of variation in response to environmental factors that affect organ size autonomously. Fluctuating asymmetry (FA) arises through stochastic perturbations in the developmental process at the molecular, chromosomal and epigenetic level [20] and is, by definition, not coordinated across the body. Implicit to the concept of FA is that, since both sides of a bilateral organism are influenced by identical genes, non-directional differences between the two sides must be environmental in origin [41]. FA can therefore be considered a reflection of environmental variation that acts at the level of individual organs (and tissues within those organs) rather than through systemic mechanisms. Our finding that FA for genital traits is the same or higher than for somatic traits suggests that genital traits do not have reduced sensitivity to environmental factors that act autonomously on organs or tissues.

Our results suggest that there are two broad classes of developmental mechanisms that regulate organ size in *Drosophila*: (1) systemic mechanisms that regulate organ and body size as a whole, for example the level of circulating growth hormone; and (2) organ autonomous mechanisms that affect the size of organs individually, for example the expression of genes that pattern individual organs. The genitalia appear to have reduced their response to the former but not the latter affectors of size (Fig. 1A). This is to be expected. The slope of an organ-body size scaling relationship captures the extent to which factors that generate variation in body size also generate variation in organ size; the evolution of hypoallometry (or hyperallometry) should therefore involve changes in the response of an organ to these factors.

How the genitalia reduce their response to systemic regulators of size is unclear but is an area of active research. For example, the developing genitalia are insensitive to changes in insulin signaling, the primary developmental mechanism through which nutrition regulates growth in all animals [42]. Changes in nutrition during development affect the level of circulating insulin-like peptides that in turn affects the rate of cell proliferation in growing tissues. Because the growth rate of the genitalia is relatively insensitive to changes in insulin-signaling, their final size is less sensitive to changes in nutrition and the genitalia are nutritionally canalized [21]. Importantly, insulin-insensitivity could also account for the genetic canalization of the genitalia and the low slope of their genetic static allometry. Genetic variation in body size has been linked to allelic variation within the insulin-signaling pathway [43]. Organs that are insensitive to changes in insulin-signaling caused by nutritional variation should also be insensitive to changes in insulin-signaling caused by genetic variation. More generally, if genetic variation in body and organ size is primarily mediated by genes involved in the environmental regulation of size, then environmental and genetic canalization may reflect the same developmental processes.

A deeper understanding of the developmental mechanisms that underlie the genetic and environmental canalization of *Drosophila* genitalia will help clarify the adaptive significance of their low allometric slope. There are a number of alternative hypotheses to account for genital hypoallometry [22]. The ‘lock-and-key hypothesis’ argues that male genitalia need to be of a particular size in order to physically fit with the female genitalia, with strong stabilizing selection for genitalia of an intermediate size [44]. The ‘one-size-fits-all’ hypothesis is similar but proposes that there is stabilizing sexual selection rather than natural selection for genitalia of an intermediate size, with females favoring males with such genitalia [44]. These models have been criticized more recently, in part because empirical studies have revealed directional selection on genital size in male water striders (*Aquarius remigis*) despite being hypoallometric to body size [27]. In response to this criticism the models have been extended from their original implications of stabilizing selection to include directional selection. Specifically, hypoallometry may result if positive directional selection on genital size is more intense for small males than large males [26].

Our data suggest that genital hypoallometry is not a consequence of stabilizing selection on genital size alone. Stabilizing selection should not only reduce the genetic variation in genital size that is correlated with variation in the size of other traits but also the genetic variation in genital size that is organ autonomous [26], [28], which we did not find. Further, stabilizing selection might also be expected to reduce the developmental instability of the genital traits [32] (but see [34]), also not supported by our data. Rather, the finding that the genitalia are only canalized with respect to genetic and environmental factors that generate systemic variation in body and organ size suggests that selection for hypoallometry has targeted the mechanisms that regulate the response of the genitalia to these factors. These mechanisms ultimately regulate the relationship between genitalia and body size.

**Figure 2:**
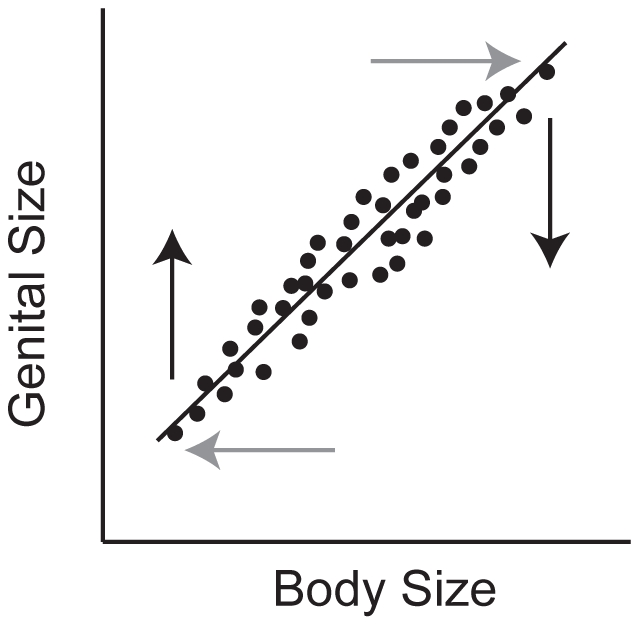
Model of a selection regime that alters the slope of the genital-body scaling relationship while maintaining genital-autonomous genetic variation. Selection is for proportionally smaller genitalia in large males and proportionally larger genitalia in small males (black arrows). Implicit to such a regime is that there is selection or constraint maintaining variation in body size (gray arrows).

What form of selection would target these mechanisms preferentially? One hypothetical selection regime favors large genitalia in small males and small genitalia in large males (Fig. 2). Such selection is not expected to reduce genital-autonomous genetic variation. This is because alleles that make the genitalia autonomously large will be selected against in large males but selected for in small males, maintaining overall genetic variation. The inverse is true for alleles that make the genitalia autonomously small. In contrast, alleles that reduce the relative sensitivity of the genitalia to systemic genetic and environmental regulators of organ size will be favored in both large and small males. Implicit to this selection regime is the assumption that variation in body and somatic trait size is maintained, either through selection or constraint (Fig. 2). Directional selection on genital size that is more negative in large males than small males, or more positive in small males than large males, should similarly target genes that influence the relationship between genitalia and body size and change the slope of their scaling relationship [26]. However, like stabilizing selection, such directional selection might be expected to also reduce organ-autonomous genetic variation in the genitalia [45], [46]. On the other hand, of all morphological traits genital traits may be most closely related to fitness. Fitness traits seem to have elevated levels of variance [47] and this may counter the effects of directional selection on organ-autonomous genetic variation in genital size.

Interestingly, the femur of the first leg of male *Drosophila*, like the genital traits, also showed low levels of total genetic variation and scaled hypoallometrically to body size. The first legs of male *Drosophila* carry the sex-combs, thought to be used for grasping the female genitalia prior to intromission [48], [49]. One hypothesis for the reduced total genetic variation of the femur therefore is that similar selective pressures are acting on the first leg and genitalia in male *Drosophila*.

In general, the slope of allometric scaling relationships is a multivariate trait that reflects variation in organ size, body size and the relationship between the two. Specifically, it describes the extent to which environmental or genetic factors influence trait size relative to body size. Implicit to the concept of allometry is that these factors should affect both trait size and body size. Theories as to how allometric slopes evolve must therefore consider selection on organ size relative to body size, rather than organ size alone. Consequently, future studies should explore how selection acts on genital size in males of varying sizes. Our prediction is that, if there is selection for hypoallometry, the strength and direction of selection on genital size will depend on the size of the male the genital is attached to.
